# Identification of *RUNX2* variants associated with cleidocranial dysplasia

**DOI:** 10.1186/s41065-019-0107-7

**Published:** 2019-09-16

**Authors:** Xueren Gao, Kunxia Li, Yanjie Fan, Yu Sun, Xiaomei Luo, Lili Wang, Huili Liu, Zhuwen Gong, Jianguo Wang, Yu Wang, Xuefan Gu, Yongguo Yu

**Affiliations:** 1 Department of Pediatric Endocrinology and Genetics, Shanghai Institute for Pediatric Research, Xinhua Hospital, School of Medicine, Shanghai Jiaotong University, Shanghai, 200092 China; 2 The Affiliated Yantai Yuhuangding Hospital of Qingdao University, Qingdao, Shandong China

**Keywords:** RUNX2, Pathogenic variant, Cleidocranial dysplasia, Targeted next-generation sequencing

## Abstract

**Background:**

Cleidocranial dysplasia (CCD) is a rare autosomal dominant disorder mainly characterized by hypoplastic or absent clavicles, delayed closure of the fontanelles, multiple dental abnormalities, and short stature. Runt-related transcription factor 2 (*RUNX2*) gene variants can cause CCD, but are not identified in all CCD patients.

**Methods:**

In this study, we detected genetic variants in seven unrelated children with CCD by targeted high-throughput DNA sequencing or Sanger sequencing.

**Results:**

All patients carried a *RUNX2* variant, totally including three novel pathogenic variants (c.722_725delTGTT, p.Leu241Serfs*8; c.231_232delTG, Ala78Glyfs*82; c.909C > G, p.Tyr303*), three reported pathogenic variants (c.577C > T, p.Arg193*; c.574G > A, p.Gly192Arg; c.673 C > T, p.Arg225Trp), one likely pathogenic variant (c.668G > T, p.Gly223Val). The analysis of the variant source showed that all variants were de novo except the two variants (c.909C > G, p.Tyr303*; c.668G > T, p.Gly223Val) inherited from the patient’s father and mother with CCD respectively. Further bioinformatics analysis indicated that these variants could influence the structure of RUNX2 protein by changing the number of H-bonds or amino acids. The experimental result showed that the Gly223Val mutation made RUNX2 protein unable to quantitatively accumulate in the nucleus.

**Conclusions:**

The present study expands the pathogenic variant spectrum of *RUNX2* gene, which will contribute to the diagnosis of CCD and better genetic counseling in the future.

## Background

Cleidocranial dysplasia (CCD; OMIM #119600) is a rare autosomal dominant disorder mainly characterised by hypoplastic or absent clavicles, delayed closure of fontanelles, multiple dental abnormalities, and short stature [1–3]. Variants in runt-related transcription factor 2 (*RUNX2*) gene (OMIM *600211) can result in haploinsufficiency of the protein and have been related to CCD [1, 2]. The *RUNX2* gene is located on chromosome 6p21.1 and encodes a transcription factor with a highly conserved Runt domain [4, 5]. The Runt domain is responsible for binding to a specific DNA motif (TG^T^/_C_GGT sequence) in the promoter region of its target genes and heterodimerization with CBFB (core-binding factor subunit beta) [6–8]. The former participates in regulating the transcription of multiple genes. The latter increases the DNA-binding affinity as well as protects and stabilizes RUNX2 against proteolytic degradation. The N-terminal side of the Runt domain links a Q/A region consisting of 23 consecutive glutamine residues followed by 17 alanine residues, which acts as a second transactivation domain [9]. The C-terminal side of the Runt domain links a PST (proline/serine/threonine)-rich region, which contains the phosphorylation sites and represents the third transactivation domain [9, 10]. The last five amino acids (VWRPY) of RUNX2 protein compose a conserved motif in all runt proteins, and functions as a transcriptional repression domain [9, 11].

RUNX2 is essential for osteoblastic differentiation and skeletal morphogenesis. In mouse models, the homozygous mutation of *RUNX2* gene blocked both intramembranous and endochondral ossification and resulted in a complete lack of bone formation [12]. The heterozygous mutation (*RUNX2*^+/−^) caused a similar phenotype to that of human CCD [13]. To date, 184 publicly available mutations in *RUNX2* gene have been deposited in the Human Gene Mutation Database (HGMD, www.hgmd.cf.ac.uk). Most of these mutations were missense and clustered in Runt domain. Additionally, nonsense mutations, insertions or deletions are also observed in the *RUNX2* gene, which are predominant within the Q/A domain or the PST domain. Although many mutations in the *RUNX2* gene have been identified in familial and sporadic cases, novel mutation is still reported recently, suggesting that mutational screening on *RUNX2* gene is far from saturation [14–19].

In the present study, we conducted genetic evaluation for a cohort of seven Chinese children with CCD by targeted high-throughput DNA sequencing or Sanger sequencing, and found seven different variants in *RUNX2* gene, including six pathogenic variants and one likely pathogenic variant. These results will contribute to the diagnosis of CCD and better genetic counseling in the future.

## Material and methods

### Genomic DNA extraction and genetic testing

A total of seven unrelated children with CCD ranging in age from 1 month to 12 years were enrolled for genetic evaluation (Table 1). Genomic DNA of probands and their family members was extracted from peripheral blood leukocytes using Lab-Aid Nucleic Acid Isolation Kit (Zeesan, China), according to the manufacturer’s instructions.

**Table 1:** Genetic detection methods and basic characteristics of seven children with CCD

Proband ID	Gender	Age	Family history	Genetic detection methods
Family_A_II1	Male	3Y	No	Inherited disease panel (Agilent) Hiseq4000(Illumina), Sanger sequencing
Family_B_II1	Female	1Y9M	No	Focused exome panel (Agilent) Hiseq2500(Illumina), Sanger sequencing
Family_C_II1	Male	9Y11 M	No	Sanger sequencing
Family_D_II1	Male	12Y	No	xGen Exome research panel v1.0 (IDT) HiSeq4000(Illumina), Sanger sequencing
Family_E_II1	Female	1 M	No	Sanger sequencing
Family_F_III1	Male	3Y	Father with CCD	xGen Exome research panel v1.0 (IDT) HiSeq4000(Illumina), Sanger sequencing
Family_G_III1	Male	6Y	Mother with CCD Uncle with CCD Grandmother with CCD	xGen Exome research panel v1.0 (IDT) HiSeq4000(Illumina), Sanger sequencing

*Y* Year, *M* Month

Among these CCD patients, five patients were firstly detected by targeted high-throughput DNA sequencing, two patients directly by Sanger sequencing (Table 1). For targeted high-throughput DNA sequencing, the preparation of sequencing library was completed using Agilent Inherited Disease panel, Agilent Focused exome panel or xGen Exome research panel v1.0 (Integrated DNA Technologies, Coralville, Iowa). Sequencing was performed on the Illumina HiSeq 2500 or 4000 (Illumina, San Diego, CA), according to the manufacturer’s instructions. Burrows-Wheeler Aligner (BWA, version 0.7.10) was used to mapping reads to the human reference genome (GRCh37/hg19). Base calling, QC analysis and coverage analysis were performed with Picard tools-1.124 and GATK software. Variants were annotated using SnpEff version 4.2. Subsequently, the following variants were filtered out: (i) variants with > 1% frequency in the population variant databases including 1000 Genomes Project, Exome Variant Server (EVS) and Exome Aggregation Consortium (ExAC) or > 5% frequency in our inhouse database (based on 150 exome datasets), (ii) intergenic and 3′/5′ untranslated region variants, none splice-related intronic and synonymous variants.

For Sanger sequencing, all exons of the *RUNX2* gene in these probands were amplified by PCR reaction. DNA sequence variants were identified by Mutation Surveyor V4.0.5 software with reference sequences (NG_008020.1).

### Variant assessment

MutationTaster (http://www.mutationtaster.org), SIFT (http://sift.jcvi.org), and PolyPhen-2 (http://genetics.bwh.harvard.edu/pph2/) were used to assess pathogenic potential of the variants [20–22]. Combined with clinical manifestation and modes of inheritance, candidate variants were validated by Sanger sequencing for all family members, and classified according to standards and guidelines of the American College of Medical Genetics and Genomics (ACMG). For the putative pathogenic or likely pathogenic variants, SWISS-MODEL (https://swissmodel.expasy.org) and Swiss-PdbViewer 4.1 software (http://spdbv.vital-it.ch/) were used to analyze the effect of these variants on protein structure [23, 24].

### Subcellular localization of the RUNX2 mutant protein

The cDNA of wide-type *RUNX2* gene was synthesized by Sangon Biotech (Shanghai) Co., Ltd., and amplified by PCR. The forward primer was 5′-GACACAGATCTCGAGATGGCATCAAACAGCCTCTTCAGC-3′ and the reverse primer was 5′-GTGTCGTCGACTGATATGGTCGCCAAACAGATTCA-3′. The PCR fragment was subcloned into pEGFP-N1 vector with the XhoI and SaII restriction sites. The *RUNX2* 668G > T (Gly223Val) mutation was introduced into pEGFP-N1 vector with wide-type RUNX2 cDNA by site-directed mutagenesis. The mutant primers were 5′-GCCTTCTGGGTTCCCGAGGTACATCTACTGTAACTTT AAT-3′, and 5′-ATTAAAGTTACAGTAGATGTACCTCGGGAACCCAGAAGGC-3′. All recombinant vectors were fully sequenced to exclude any additional mutations. The empty vector acting as a negative control (NC) and pEGFP-N1 vectors bearing wild type (WT) and mutant (Mut) were transfected into U2OS cells by using lipofectamine 2000 (Invitrogen). The cells were visualized and photographed (magnification 10X and 40X) with a fluorescent microscope (Olympus IX73, Japan).

## Results

### Clinical features of CCD children

All children underwent a clinical evaluation and were diagnosed as CCD by an experienced pediatrician. The clinical features of these patients including two female and five male patients were summarized in Table 2. Besides the clavicle and skull dysplasia, short stature, scoliosis, enamel hypoplasia, delayed eruption of deciduous teeth, low nasal bridge, delayed mineralization of pubic bone, broad femoral head with short femoral neck, hypoplastic iliac wing, syringomyelia and special faces were also observed in CCD children. Furthermore, hypertelorism was observed in all CCD children, except Family_A_II1. Supernumerary teeth, retention cysts and long second metacarpal were observed in all CCD children, except Family_A_II1 and Family_B_II1.

**Table 2:** Comparison of clinical features of CCD children with different *RUNX2* gene variant

Clinical synopsis	Family_A_II1 (c.577C > T)	Family_B_II1 (c.574G > A)	Family_C_II1 (c.673C > T)	Family_D_II1 (c.722_725delTGTT)	Family_E_II1 (c.231_232delTG)	Family_F_III1 (c.909C > G)	Family_G_III1 (c.668G > T)
GROWTH							
*Height*							
Short stature	√	√	√	√	√	√	√
HEAD & NECK							
*Head*							
Delayed fontanelle closure	√	√	√	√	√	√	√
Parietal bossing	√	√	√	√	√	√	√
Anterior fontanelle open in adults							
*Face*							
Frontal bossing	√	√	√	√	√	√	√
Metopic groove	√	√	√	√	√	√	√
Midface hypoplasia	√	√	√	√	√	√	√
Micrognathia	√	√	√	√	√	√	√
*Ears*							
Deafness							
*Eyes*							
Hypertelorism		√	√	√	√	√	√
*Nose*							
Low nasal bridge	√	√	√	√	√	√	√
*Mouth*							
Cleft palate							
Narrow, high-arched palate							
*Teeth*							
Delayed eruption of deciduous teeth	√	√	√	√	√	√	√
Delayed eruption of permanent teeth							
Supernumerary teeth			√	√	√	√	√
Retention cysts			√	√	√	√	√
Enamel hypoplasia	√	√	√	√	√	√	√
RESPIRATORY							
*Airways*							
Respiratory distress in early infancy							
CHEST							
*External Features*							
Narrow thorax	√	√	√	√	√	√	√
Abnormal facility in opposing the shoulders	√	√	√	√	√	√	√
*Ribs Sternum Clavicles & Scapulae*							
Small scapula							
Hypoplastic clavicles	√	√	√	√	√	√	√
Aplastic clavicles							
Short ribs							
Cervical ribs							
SKELETAL							
Osteosclerosis							
Increased bone fragility	√	√	√	√	√	√	√
*Skull*							
Wormian bones	√	√	√	√	√	√	√
Bossing of frontal bone	√	√	√	√	√	√	√
Bossing of occipital bone							
Bossing of parietal bone	√	√	√	√	√	√	√
Calvarial thickening							
Absent frontal sinuses							
Absent paranasal sinuses							
Hypoplastic frontal sinuses							
Hypoplastic paranasal sinuses							
Large foramen magnum							
*Spine*							
Spondylolysis							
Spondylolisthesis							
Scoliosis	√	√	√	√	√	√	√
Kyphosis							
*Pelvis*							
Wide pubic symphysis							
Delayed mineralization of pubic bone	√	√	√	√	√	√	√
Broad femoral head with short femoral neck	√	√	√	√	√	√	√
Coxa vara							
Hypoplastic iliac wing	√	√	√	√	√	√	√
*Hands*							
Brachydactyly							
Long second metacarpal			√	√	√	√	√
Short middle phalanges of second and fifth fingers							
Cone-shaped phalangeal epiphyses							
NEUROLOGIC							
*Peripheral Nervous System*							
Syringomyelia	√	√	√	√	√	√	√

### Genetic testing

**Fig. 1:**
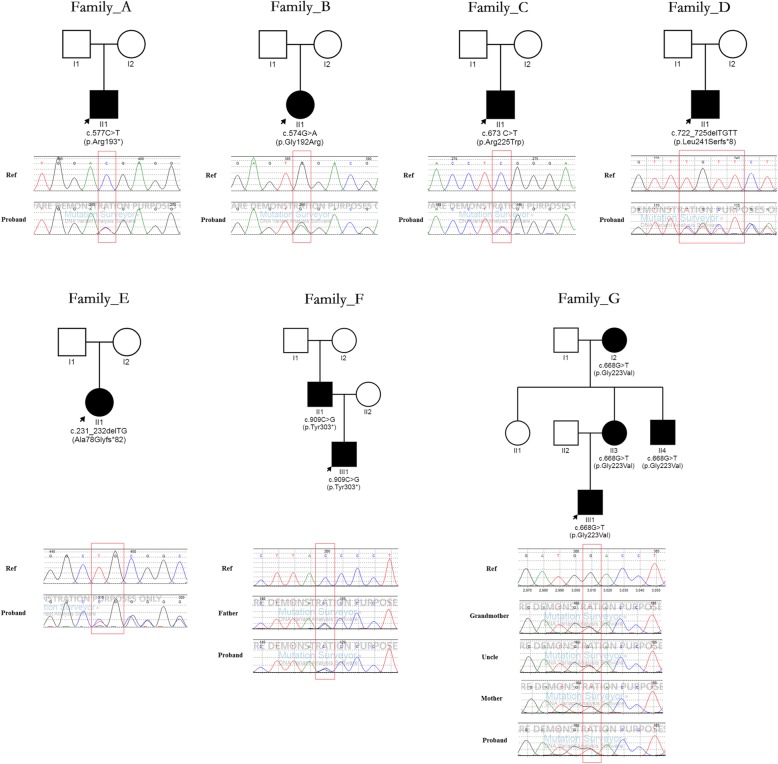
The pedigree of the family and Sanger sequence chromatograms of *RUNX2* gene variants (The black arrow indicates the proband)

**Fig. 2:**
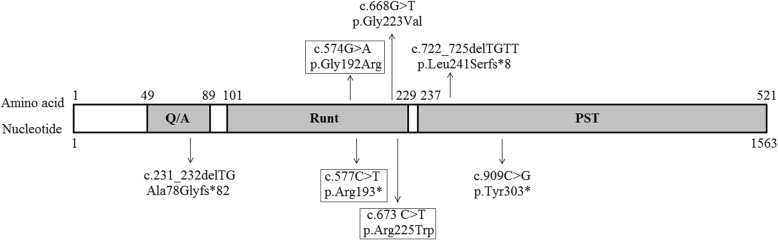
Relative positions of *RUNX2* gene variants identified in seven children with CCD (Variants in the box indicates the reported variants)

All patients carried a *RUNX2* variant, totally including four novel variants and three reported variants (Figs. 1, 2 and Table 3). Among the seven variants, there were two pathogenic missense variants (c.574G > A, p.Gly192Arg; c.673 C > T, p.Arg225Trp), one likely pathogenic missense variant (c.668G > T, p.Gly223Val), two pathogenic frameshift variants (c.722_725delTGTT, p.Leu241Serfs*8; c.231_232delTG, Ala78Glyfs*82), and two pathogenic stop-gain variants (c.577C > T, p.Arg193*; c.909C > G, p.Tyr303*). The analysis of the variant source showed that all variants were de novo except the two variants (c.909C > G, p.Tyr303*; c.668G > T, p.Gly223Val). The former variant was inherited from the patient’s father with CCD, who carried a de novo heterozygous *RUNX2* variant (c.909C > G, p.Tyr303*). The latter variant was inherited from the patient’s mother with CCD, who carried a maternal inherited and heterozygous *RUNX2* variant (c.668G > T, p.Gly223Val).

**Table 3:** Summarization of *RUNX2* gene variants in seven children with CCD

Proband ID	Variant location	Variant type	Variant source	Literature report	Bioinformatic prediction			ACMG classification
					MutationTaster	SIFT	PolyPhen-2	
Family_A_II1	NM_001024630.3: c.577C > T, p.Arg193* (Het)	Stopgain	De novo	Hum Mol Genet. 1999;8 (12):2311–6.	Disease causing	NA	NA	Pathogenic
Family_B_II1	NM_001024630.3: c.574G > A, p.Gly192Arg (Het)	Missense	De novo	J Hum Genet. 2005;50 (12):679–83.	Disease causing	Damaging	Probably damaging	Pathogenic
Family_C_II1	NM_001024630.3: c.673 C > T, p.Arg225Trp (Het)	Missense	De novo	Am J Hum Genet. 1999;65 (5):1268–78.	Disease causing	Damaging	Probably damaging	Pathogenic
Family_D_II1	NM_001024630.3: c.722_725delTGTT, p.Leu241Serfs*8 (Het)	Frameshift	De novo	_	Disease causing	NA	NA	Pathogenic
Family_E_II1	NM_001024630.3: c.231_232delTG, Ala78Glyfs*82 (Het)	Frameshift	De novo	_	Disease causing	NA	NA	Pathogenic
Family_F_III1	NM_001024630.3: c.909C > G, p.Tyr303* (Het)	Stopgain	Paternal inheritance	_	Disease causing	NA	NA	Pathogenic
Family_G_III1	NM_001024630.3: c.668G > T, p.Gly223Val (Het)	Missense	Maternal inheritance	_	Disease causing	Damaging	Probably damaging	Likely pathogenic

*NA* Not available; * the stop codon

### The effect of the RUNX2 variants on protein structure

Among these variants, there were three variants changing the number of H-bonds in RUNX2 protein, including two variants increasing H-bonds (c.574G > A, p.Gly192Arg; c.668G > T, p.Gly223Val) and one variant decreasing H-bonds (c.673 C > T, p.Arg225Trp). In addition, there were four variants (c.722_725delTGTT, p.Leu241Serfs*8; c.231_232delTG, Ala78Glyfs*82; c.577C > T, p.Arg193*; c.909C > G, p.Tyr303*) decreasing the number of amino acids in RUNX2 protein.

### Subcellular localization of the RUNX2 mutant protein

**Fig. 3:**
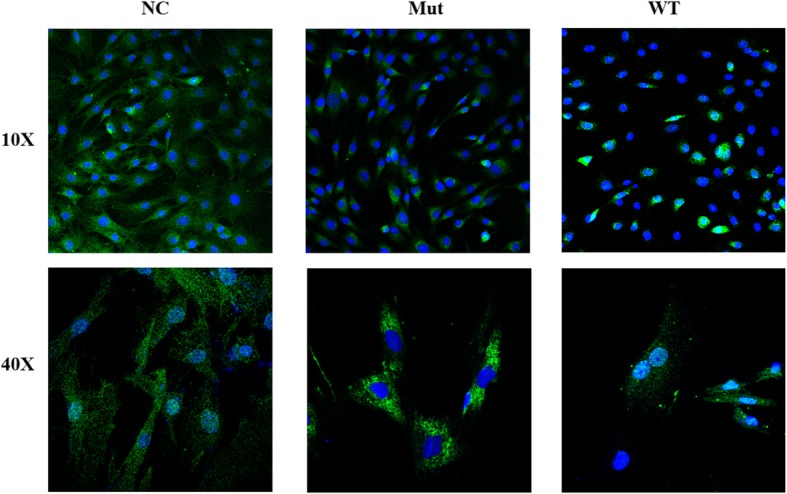
Subcellular localization of the RUNX2 mutant protein (NC, Mut, WT indicate control GFP, mutant Gly223Val RUNX2 and wild-type RUNX2, respectively. Magnification 10X and 40X)

To further explore the function of the missense mutation (c.668G > T, p.Gly223Val) not reported, the wide-type and mutant RUNX2 proteins binding green fluorescent protein (GFP) were constructed and transiently transfected into human osteosarcoma U2OS. The result showed that the Gly223Val mutation could affect the subcellular distribution of RUNX2 protein and made RUNX2 protein unable to quantitatively accumulate in the nucleus (Fig. 3).

## Discussion

CCD is a skeletal dysplasia that represents a continuum of clinical findings ranging from classical CCD (dental abnormalities, hypoplastic or aplastic clavicles, and delayed closure of the cranial sutures) to mild CCD to isolated dental anomalies without other skeletal features. To date, no formal clinical diagnostic criteria for CCD have been established. Due to CCD inherited in an autosomal dominant manner, each child of an individual with CCD has a 50% chance of inheriting the pathogenic variant. If the pathogenic variant in the family is known, prenatal diagnosis for pregnancies at increased risk will be possible. Many kinds of molecular testing approaches, including single-gene testing, karyotype analysis and a multigene panel, can be currently used to detect the variants leading to CCD. For single-gene testing, sequence analysis of *RUNX2* gene is performed first and followed by gene-targeted deletion/duplication analysis if no pathogenic variant is identified. For karyotype analysis, if *RUNX2* testing is not diagnostic and strong suspicion persists in an individual with CCD features who also has multiple congenital anomalies and/or developmental delay, a karyotype analysis may be considered to evaluate complex chromosome rearrangements or translocations that involve *RUNX2* locus but do not result in *RUNX2* copy number changes [25, 26]. In addition, a multigene panel that includes *RUNX2* and other genes of interest may also be considered.

**Fig. 4:**
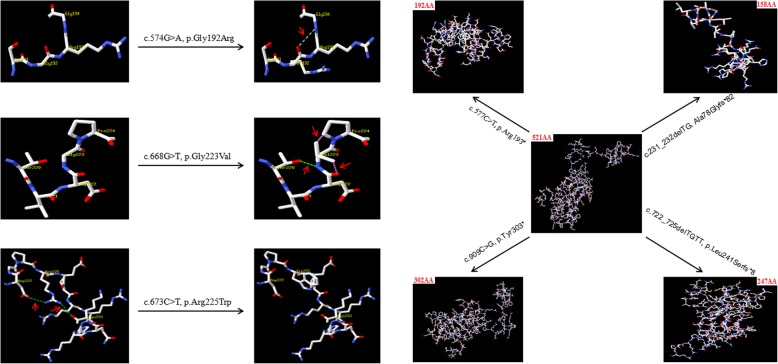
The effect of *RUNX2* gene variants on protein structure (The red arrow indicates the H-bonds)

In the present study, we utilized targeted high-throughput DNA sequencing or Sanger sequencing (single-gene testing) techniques to analyze genetic variants in seven CDD children, and found seven different variants in *RUNX2* gene, including four novel variants (c.722_725delTGTT, p.Leu241Serfs*8; c.231_232delTG, Ala78Glyfs*82; c.909C > G, p.Tyr303*; c.668G > T, p.Gly223Val) and three reported variants (c.577C > T, p.Arg193*; c.574G > A, p.Gly192Arg; c.673 C > T, p.Arg225Trp) [27–29], which were all located in the transactivation region (Fig. 2). The bioinformatics analysis indicated that these variants were disease-causing, damaging and/or probably damaging variants. According to ACMG, six variants (c.574G > A, p.Gly192Arg; c.673 C > T, p.Arg225Trp; c.577C > T, p.Arg193*; c.722_725delTGTT, p.Leu241Serfs*8; c.231_232delTG, Ala78Glyfs*82; c.909C > G, p.Tyr303*) were classified as pathogenic variants, and one variant (c.668G > T, p.Gly223Val) as likely pathogenic variant. In addition, all variants were de novo except the following two variants: c.909C > G, p.Tyr303* and c.668G > T, p.Gly223Val*.* Thereinto the former variant (c.909C > G, p.Tyr303*) was inherited from the patient’s father, who is also a CCD patient carried a de novo heterozygous *RUNX2* variant. The clinical features of the father included short stature and CCD, which were very similar to those of his 3-year-old son. The latter variant (c.668G > T, p.Gly223Val) was inherited from the patient’s mother with CCD, who carried a maternal inherited and heterozygous *RUNX2* variant. Both of them also showed similar clinical phenotypes, such as short stature and CCD. By summarizing *RUNX2* variants in HGMD and the current study, we found nine variant types, such as missense/nonsense, splicing, small deletions/insertions, gross insertions/duplications. Thereinto missense/nonsense variant was the most common variant type of *RUNX2* gene (Table 4). A single amino acid (Gly) substitution at position 332 in RUNX2 protein was found not only in our lab (c.668G > T, p.Gly223Val), but also in Ott’ s study (c.667G > A, p.Gly223Arg) [1]. In addition, protein structure prediction showed that these variants could change the number of H-bonds or amino acids in RUNX2 protein (Fig. 4), suggesting that these variants played an important role in regulating the effective structure and function of RUNX2 protein. The experimental result showed that Gly223Val mutation, located in nuclear localization sequence (NLS) [29, 30], could affect the subcellular distribution of RUNX2 protein. The mutation made RUNX2 protein unable to quantitatively accumulate in the nucleus.

**Table 4:** Summarization of *RUNX2* gene variants in the HGMD and current study

Variant type	Number of variants (%)		
	HGMD	The current study	Total
Missense/nonsense	77 (41.8%)	5 (71.4%)	82 (42.9%)
Splicing	11 (6.0%)	–	11 (5.8%)
Small deletions	44 (23.9%)	2 (28.6%)	46 (24.1%)
Small insertions	22 (12.0%)	–	22 (11.5%)
Small indels	2 (1.1%)	–	2 (1.0%)
Gross deletions	17 (9.2%)	–	17 (8.9%)
Gross insertions/duplications	5 (2.7%)	–	5 (2.6%)
Complex rearrangements	4 (2.2%)	–	4 (2.1%)
Repeat variations	2 (1.1%)	–	2 (1.0%)

In conclusion, the present study reveals some novel genetic causes of CDD, which not only expands the pathogenic variant spectrum of *RUNX2* gene but also will contribute to the diagnosis of CCD and better genetic counseling in the future.

## Data Availability

The datasets used and/or analysed during the current study are available from the corresponding author on reasonable request.

## Abbreviations

ACMG: American College of Medical Genetics and Genomics
CCD: Cleidocranial dysplasia
EVS: Exome Variant Server
ExAC: Exome Aggregation Consortium
RUNX2: Runt-related transcription factor 2
